# Does higher endorsement with collaboration lead to better performance on collaborative problem solving? An explanatory item response approach to cross-cultural comparisons

**DOI:** 10.3389/fpsyg.2024.1468533

**Published:** 2024-11-08

**Authors:** Chang Lu, Okan Bulut

**Affiliations:** ^1^School of Education, Shanghai Jiao Tong University, Shanghai, China; ^2^Centre for Research in Applied Measurement and Evaluation, Faculty of Education, University of Alberta, Edmonton, AB, Canada

**Keywords:** attitudes towards collaboration, collaborative problem solving, cultural differences, explanatory item modeling, attitude and performance

## Abstract

**Introduction:**

Collaborative problem solving (CPS) is an essential competency in the 21st century. However, the understanding of how cultural background shapes individuals’ collaboration awareness and its relationship with collaborative problem-solving skills are underexplored by psychometrics.

**Methods:**

This study employs Explanatory Item Response Modeling to examine the impact of cultural orientations on students’ endorsement with collaboration in PISA 2015.

**Results:**

Results show that students endorsed valuing teamwork more than valuing relationships. Western countries with individualist cultures generally demonstrated higher endorsement with collaboration than Eastern countries with collectivist cultures. China has the highest collaboration endorsement, followed by the US, Canada, Korea, and Japan. In contrast, Japan has the highest CPS assessment scores, followed by Korea, Canada, the US, and China.

**Discussion:**

Findings revealed that students from collectivist cultures do not have higher endorsements of collaboration compared with students from individualist cultures. Also, higher endorsement of collaboration does not necessarily lead to better success in the CPS assessment. Further research is needed to understand the gap between students’ attitudes towards collaboration and their achievement based on cultural values and schools’ CPS training.

## Introduction

Collaborative problem solving (CPS) is regarded as one of the core competencies in the 21st century (von Davier and Halpin, 2013; Griffin and Care, 2014). CPS is the process of “approaching a problem responsively by working together and exchanging ideas” to achieve mutual goals by two or more parties (Hesse et al., 2015). It is often conceptualized as a highly loaded skill that systematically combines problem-solving competencies and communicative skills in collaboration and teamwork situations. This process requires an understanding of the context, establishing, and making use of knowledge, as well as endeavoring individual efforts and contributing to the group to achieve a specific goal (Griffin et al., 2013; Hilse et al., 2014).

During the last decade, CPS skills have been gaining attention in education, and an increasing number of curricula and assessment programs have included CPS as part of their agenda (OECD, 2017). In 2015, CPS tasks were first integrated into the twenty-first-century skills framework as a key competency and were introduced as a principal assessment section by the Assessment and Teaching of 21st-Century Skills (ATC21S) and the Organization for Economic Co-operation and Development (OECD) boards (Griffin and Care, 2014). Since then, many studies have been conducted on exploring potential factors that influence CPS and searching for effective tools to teach and assess CPS skills (Kirschner et al., 2012; Rosen and Rimor, 2016; Stefik et al., 1987).

Concurrently, discussions are also ongoing to understand CPS from different perspectives, especially on factors that are attributed to the success of collaboration (Griffin et al., 2013; Sycara et al., 2013). Previous studies suggest that confounding factors such as gender, cultural background, and language proficiency can influence the process as well as the outcomes of collaboration (Chang et al., 2017; Hao et al., 2017). However, cross-national empirical studies focusing on the relationship between students’ academic achievement and their attitudes towards CPS attitudes lagging.

The present study aims to examine the impact of cultural orientations on individuals’ awareness of collaboration from five representative countries (i.e., China, Japan, Korea, the United States, and Canada). The collaboration scale from Program for International Student Assessment (PISA) 2015 (OECD, 2017) was used as an instrument to measure levels of endorsement and awareness of collaboration. Explanatory item response modeling (EIRM) was used to analyze students’ responses to the items on the collaboration scale based on item-level and person-level covariates and to reveal students’ potential attitudinal differences towards collaboration on two dimensions (i.e., valuing relationships and valuing teamwork). This study was guided by the following research questions:

1) To what extent do students endorse with valuing relationships versus valuing teamwork?
2) To what extent do students from the different cultural background vary on their endorsement with valuing relationships and valuing teamwork?
3) Does higher endorsement with collaboration associate with better performance on collaborative problem solving?

The significance of the present study is threefold. Theoretically, we examined the impact of cultural orientations on students’ attitudes towards collaboration through a validated, large-scale international survey. Results can be used for planning future research on fostering attitudes towards collaboration and CPS competency. Methodologically, the present study is the first study that employs EIRM to analyze the polytomous responses in a large-scale survey. The explanatory item response models provide rich information on how students’ responses in the collaboration scale differed based on both item properties and student characteristics. Pedagogically, findings facilitate educators and policymakers make informed decisions on teaching and learning of collaborative problem-solving.

## Literature review

### Importance of endorsement with collaborative problem solving

Collaboration is defined as a “coordinated, synchronous activity that is the result of a continued attempt to construct and maintain a shared conception of a problem” (Roschelle and Teasley, 1995, p. 70). CPS is often dichotomized into the social domain of collaboration skills and the cognitive domain of problem-solving skills. The problem-solving process involves cognitive abilities including task regulation, perspective-taking, and reflecting. The collaboration process is defined as the activity of working together as a team instead of individuals to achieve a common goal (Care et al., 2015). The process of collaboration can be further divided into three main aspects: participation, perspective-taking, and social regulation. Specifically, participation is defined as an individual’s willingness to offer and share information and thoughts, and the extent of their involvement as part of a team. Perspective-taking skills, as another dimension of exchange information, refers to an individual’s ability to internalize others’ opinions and make adaptations or adjustments for their purposes (Dehler et al., 2011).

In the Framework for Teachable Collaborative Problem Solving Skills, one of the key factors that ensures a successful collaboration is shared mental representation, that is, the participant’s mental representation should be aligned to other group members’ mental spaces within the same group (Hesse et al., 2015). Many studies have shown that participants usually demonstrate a higher performance of collaboration or collaborative problem solving when higher levels of shared mental representations are achieved (Klimoski and Mohammed, 1994). This type of alignment could take effect in each step of collaboration including goal setting, plan execution, communication, and progress evaluation. In this sequence of joint actions, participant’s awareness, or willingness to collaborate, plays a crucial role in positively fostering the successful fulfillment of the CPS task.

### Endorsement with CPS shaped by cultural orientation

Given the importance of endorsement with collaboration to CPS skills, many theoretical, as well as empirical studies, were dedicated to exploring factors that influence individual’s attitudes towards collaboration in different domains (e.g., Bollen et al., 2019; Cradock-Henry et al., 2017; Mattessich and Monsey, 1992). Among many causes, it is found that culture plays an important role in shaping individuals’ awareness of collaborations and cognitive development through social interactions (Patel et al., 2012). Culture can be referred to as national or regional culture, which serves as a vital factor that shapes the collective programming of human minds and behavior patterns, and often is represented by a set of values or beliefs about the willingness to communicate, openness to knowledge sharing, and trustfulness of interpersonal interaction (Hofstede, 2011).

To date, several studies were conducted to define culture and to investigate how culture steers individuals’ social and cognitive behaviors at large. Hofstede’s multi-dimensional model (Hofstede, 2011), a seminal framework in cross-cultural psychology, assesses national cultures across six dimensions: Power Distance, Uncertainty Avoidance, Individualism versus Collectivism, Masculinity versus Femininity, Long Term Orientation, and Indulgence versus Restraint. Originating from an extensive survey spanning over 50 countries and 100,000 participants, the model interprets cultural differences and their impact on societal behaviors and values. It postulates that these dimensions shape not just ideologies but also the collective behaviors within a society, making human actions predictable to an extent.

The impact of these cultural dimensions, particularly Individualism versus Collectivism, has been profound on collaborative practices and problem-solving, as subsequent studies indicate the profound influence of a society’s orientation on individuals’ approach to group tasks (Hofstede, 2011). These studies reveal that individualists prioritize personal goals and task achievement, often above group harmony, while collectivists tend to value group cohesion and interpersonal relationships, potentially at the expense of task outcomes. This cultural orientation influences not only personal relationships and work dynamics but also educational approaches, particularly in collaborative learning settings (e.g., Arieli and Sagiv, 2018; Arpaci et al., 2018; Zhang and Yin, 2019).

### Collaborative climates in China, Japan, Korea, US, and Canada

Hofstede’s studies utilized the World Values Survey to measure cultural values across 76 countries, introducing the Individualism vs. Collectivism (IDV) index. They found a link between a country’s wealth, geographical location, and its level of individualism, with richer Western countries tending towards individualism and Eastern countries towards collectivism. The U.S., Australia, the UK, and Canada scored high on individualism, while East Asian countries like Korea and China scored low. Subsequent research has continued to explore these cultural dimensions in representative nations. Chen (2000) conducted a comparative study and claimed that Chinese children grow up in a collectivist culture, and thus their psychological and cognitive development is different from children from individualist countries such as the United States. In terms of behavioral representations, Asian societies often display shyness, self-restraint, and high levels of socialization, whereas North American societies encourage self-assertion and personal independence. Oyserman et al. (2002) meta-analysis found that Canada and the U.S. are among the world’s most individualistic societies, valuing individual rights over social ties. However, compared to Japan and Korea, these differences in individualism versus collectivism are not as pronounced in certain behaviors. Only China distinctly favored collectivism, which has implications for understanding social behaviors in collaboration and conflict resolution across cultures.

### Explanatory item response modeling

Item response theory (IRT) models are commonly adopted tools to analyze survey responses. Traditional IRT models are widely used for item parameter estimation but often criticized for failing to incorporate additional variables that are likely to influence responses to the items. Unlike traditional IRT models, explanatory IRT models allow researchers to use both item-level (i.e., item properties) and person-level (i.e., student characteristics) when estimating item parameters and latent trait levels (De Boeck and Wilson, 2004). EIRM has originated from the generalized linear mixed modeling (GLMM) framework in which items (i.e., repeated measures) are assumed to be nested within students (i.e., random effects) within a multilevel structure. This approach enables the use of explanatory variables (i.e., covariates) at the item and person levels to predict student responses in the same model. To date, many studies have applied EIRM to a wide range of areas, including differential item functioning (French and Finch, 2010), contextual effect detection (Randall et al., 2011), and proficiency scaling (Hartig et al., 2012). However, these studies employed EIRM to model the effects of item-level and person-level variables using dichotomous item responses (i.e., correct or incorrect). To date, only several studies have utilized the EIRM approach to model polytomous responses, survey items based on a Likert scale.

Tuerlinckx and Wang (2004) first attempted to analyze a publicly-available verbal aggression dataset using two polytomous IRT models including the Rating Scale Model (RSM; Andrich, 1978) and Partial Credit Model (PCM; Masters, 1982) with item-level and person-level covariates. In their study, the explanatory form of PCM (EPCM) can be formulated as the log-odds of choosing response *j* over (*j* − 1) on item *i* for person *n*:


(1) $$\log\left(\frac{P_{nij}}{P_{ni\left(j-1\right)}}\right)=Z_{nij}\theta_{n}-X_{nij}^{\prime }\delta_{i}+\tau_{ij}$$

where $\theta_{n}$ represents the latent trait of person *n*, $Z_{nij}$ is a matrix of fixed and random effects related to the latent trait $\theta_{n}$, $\delta_{i}$ is the threshold parameter between the first (*j* = 0) and second (*j* = 1) response categories for item *i*, $X_{nij}^{\prime }$ is the matrix of fixed and random effects related to individual items, and $\tau_{ij}$ is the matrix of the distances between the first threshold parameter and the other threshold parameters for item *i*. Tuerlinckx and Wang (2004) model allowed the estimation of not only item threshold parameters but also the effects of covariates related to items and persons. However, the model could only estimate the effects of item-related covariates on the first threshold parameter. That is, the model contains no explanatory variables to describe the common variability beyond the initial threshold parameter.

Stanke and Bulut (2019) used the same verbal aggression dataset and estimated four polytomous IRT models including RSM, PCM, EPCM, and cross-classified PCM with a new parameterization that enables the estimation of the effects of explanatory variables on both the first threshold parameter and the subsequent threshold parameters. That is, the model can account for the variability in all of the threshold parameters based on item-level covariates. In Equation 2, the log-odds of choosing response *j* over (*j* − 1) for item *i* for person *n* can be calculated as:


(2) $$\log\left(\frac{P_{nij}}{P_{ni\left(j-1\right)}}\right)=Z_{nij}\theta_{n}-\left(X_{nij}^{\prime }\delta_{i}+W_{nij}^{\prime }\tau_{ij}\right)$$

where $W_{nij}^{\prime }$ is a matrix of fixed and random effects related to the threshold parameters and the remaining elements are the same as those defined for Equation 1. Compared to Tuerlinckx and Wang (2004) approach, this method is more flexible in terms of explaining how all threshold parameters differ based on item-level covariates. In this study, Stanke and Bulut (2019) approach was employed to examine the impact of cultural orientations on attitudes towards collaboration. Students’ responses to the survey items in the Collaboration Scale of PISA 2015 were analyzed based on item-level (item type: valuing relationships vs. teamwork) and person-level (country type: individualism vs. collectivism) covariates.

### The present study

To sum, the impact of culture on individuals’ attitudes towards collaboration revealed by previous studies suggests that the cultural factor provides the fundamental contexts for arousing awareness of collaboration and promoting individuals’ psychological engagement in collaborative tasks (Hofstede, 2011). Therefore, in the present study, we make two hypotheses: (1) students from collectivist cultures will have a higher endorsement of collaboration compared with students from individualistic cultures and (2) higher endorsement of collaboration will lead to better success in the CPS assessment.

## Methods

### Sample

The sample of the current study consists of students from five countries that participated in PISA 2015: China (*n* = 9,841), Japan (*n* = 6,647), Korea (*n* = 5,581), Canada (*n* = 20,058), and the United States (*n* = 5,712).

In total, 47,839 students took both the CPS assessment and the Collaboration Scale in the selected countries. The demographic information of the students and their CPS assessment scores from the five countries reported by OECD (2017) are shown in Table 1. To prepare the dataset, we first detected the missing values and found that the variables Country ID and Gender contained no missing data, whereas students’ responses to Item 1—Item 8 contained missing responses. We calculated that the proportion of missing data was lower than 5% (about 2%). Therefore, we deleted the samples with missing data and conducted further analyses.

**Table 1:** Students’ demographic characteristics by countries.

Country	Mean CPS Score	Total *n*	*n* by Gender	
China	496	9,841	Male	5,159
			Female	4,682
Japan	552	6,647	Male	3,338
			Female	3,309
Korea	538	5,581	Male	2,912
			Female	2,669
US	520	5,712	Male	2,858
			Female	2,854
Canada	535	20,058	Male	10,036
			Female	10,022

### Overview of collaboration scales in PISA 2015

PISA is a triennial assessment of competencies and attitudes that aims to measure “how well 15-year-old students approaching the end of compulsory schooling are prepared to meet the challenges of today’s knowledge societies” (OECD, 2017). Covering nearly half a million students in 2015, PISA uses a two-hour test on core subjects and a questionnaire suite. It has probed problem-solving since 2003, with a shift to computer-based assessment in 2012, defining it as cognitive processing to resolve non-obvious problems. In 2015, PISA expanded to include collaborative problem-solving (CPS), assessing individuals’ capacity to pool knowledge and efforts in a group problem-solving context. It also introduced the Collaboration Scale, assessing students’ attitudes towards valuing relationships and teamwork.

The Collaboration Scale measures students’ self-report levels of “establishing and maintaining shared understanding,” “taking appropriate action to solve the problem,” and “establishing and maintaining team organization” (OECD, 2017, p. 44). There were eight items from two domains of collaboration which measures students’ attitudes towards valuing relationships and measuring students’ attitudes towards teamwork, which are presented in Table 2. In the present study, we calculated the reliability indices (Cronbach’s Alpha) of the two scales among the selected countries and revealed that the reliability for scale of attitudes towards valuing relationships (Item1—Item4) is 0.7, and the reliability for scale of attitudes towards valuing teamwork (Item5—Item8) is 0.8, indicating satisfactory internal reliabilities of the two scales. Moreover, we also examined the construct validity of the two scales using Confirmatory Factor Analysis. The model yielded satisfactory model fit indices, with CFI = 0.94, TLI = 0.92, AGFI = 0.96, RMSEA = 0.08, and SRMR = 0.08. The standardized factor loadings of the eight items in the two scales are presented in Table 2.

**Table 2:** Items in the disposition of collaboration and teamwork blocks.

Item	Statement	Factor loading	*p*
Valuing relationship			
Item1	I prefer working as part of a team to working alone.	0.63	<0.001
Item2	I am a good listener.	0.40	<0.001
Item3	I enjoy seeing my classmates be successful.	0.55	<0.001
Item4	I take into account what others are interested in.	0.57	<0.001
Valuing teamwork			
Item5	I find that teams make better decisions than individuals.	0.68	<0.001
Item6	I enjoy considering different perspectives.	0.53	<0.001
Item7	I find that teamwork raises my own efficiency.	0.75	<0.001
Item8	I enjoy cooperating with peers.	0.76	<0.001

### Item-level and person-level covariates

To address the research questions, the effects of two explanatory variables were analyzed using EPCM. The item-level covariate *item type* is a two-way factor that explains whether an item measures attitudes towards valuing relationships or teamwork. Two sets of person-level covariates were used to code the countries based on the cultural orientations represented in Hofstede’s culture dimensions and their country identities. More specifically, *country type* refers to whether the selected country has an Eastern collectivist culture or a Western individualist culture; and *country ID* represents the identity of the selected country.

#### Item type

To explore the effect of item-level differences, we categorized items 1 to 4 as item type = 0 (valuing relationships), and items 5 to 8 as item type =1 (valuing teamwork). The effect of item type will be explored in EPCM as an item-level covariate.

#### Country type

Two categories of country type were created on the dimension of collectivism vs. individualism. The US and Canada were coded as individualist Western countries (country type = 1); and China, Korea, and Japan were coded as collectivist Eastern countries (country type = 0).

#### Country ID

Country ID was introduced in the EPCM to account for the variability in attitudes towards collaboration attitudes across the selected countries. We coded China as Country 1, Korea as Country 2, Japan as Country 3, Canada as Country 4, and the US as Country 5.

### Data analysis

#### Partial credit model

The baseline model PCM was used to understand the psychometric properties. PCM yields the estimate of the first threshold parameter and the distances between the first threshold parameter and the subsequent threshold parameters. The goal of estimating PCM prior to explanatory IRT models was to review the threshold parameters of the items in the Collaboration Scale without considering the effects of any item-level and person-level covariates.

#### Explanatory partial credit model

To answer the four research questions of this study, a weighted version of EPCM was used to account for the total variance based on an item-level variable *item type*, and two person-level variables *country type* and *country ID*. PISA 2015 adopted a two-stage stratified sampling design with country as the first layer and schools as the second layer. To prevent misleading results caused from the unbalanced sample sizes, the Country Weight variable in the dataset was introduced in the EPCM to weight students’ responses of collaboration scales as instructed by the technical report of PISA 2015 for country-wise comparisons (OECD, 2013). The log-odds of choosing response *j* over (*j* − 1) on item *i* for person *n* in the weighted EPCM can be calculated by:


(3) $$\sum_{m=1}^{n}d_{m}\log\left(\frac{P_{nij}}{P_{ni\left(j-1\right)}}\right)=\sum_{m=1}^{n}d_{m}\left(Z_{nij}\theta_{n}+X_{nij}^{\prime }\delta_{i}+W_{nij}^{\prime }\tau_{ij}\right)$$

where $\theta_{n}$ represents students’ attitudes of collaboration where higher values indicate higher levels of positive attitudes towards collaboration, $Z_{nij}$ is the matrix of the person-level predictors (i.e., country type and country ID), $\delta_{i}$ is the estimate of the first threshold parameter between the response categories of “*Strongly Disagree*” and “*Disagree*” for item *i*, $X_{nij}^{\prime }$ is the matrix of the item-level predictor (i.e., item type) for the first threshold, $\tau_{ij}$ represents the distances between the first threshold parameter and the thresholds for the subsequent response categories (i.e., “*Disagree*” to “*Agree*” and “*Agree*” to “*Strongly Agree*”), and $W_{nij}^{\prime }$ is the matrix of the item-level predictor for the thresholds of the subsequent response categories. Equation 3 also includes a vector of survey weights, $d_{m}$, for each country $\left(m=1,2,3,4,5\right)$ to correct for the impact of sampling error as instructed in the technical report of PISA 2015 (OECD, 2017). The use of survey weights makes the sample of students in the PISA 2015 database representative of the population of middle school students from the selected countries.

#### Model summary

In the present study, five models were fitted to the weighted EPCM to estimate the effect of item-level covariates and person-level covariates. Equation 4 summarizes the first model that include item-level covariate only. More specifically, $\theta_{n}$ is calculated as the student *n*’s general attitudes towards collaboration, $\delta_{1}$ and $\delta_{2}$ are the estimates of the first threshold locations (from “Strongly Disagree” to “Agree”) for *Item Type 0: valuing relationship* and *Item Type 1: valuing teamwork*, respectively, $\tau_{11}$ and $\tau_{12}$ are the estimates of distances between step thresholds (from “Disagree” to “Agree” and from “Agree” to “Strongly Agree”) for *Item Type 0: valuing relationship*, $\tau_{21}$ and $\tau_{22}$ are the estimates of distances between step thresholds for *Item Type 1: valuing teamwork*, and $\varepsilon_{j}$ is the error term. In addition, $w_{m}$ is the weight index assigned with respect to student *n*’s country identity.


(4) $$\begin{array}{l} \;w_{m}\left[\begin{array}{l} \log\left(\frac{P_{n\left(\text{Strongly}\;\text{Disagree}\right)}}{P_{n\left(\text{Disagree}\right)}}\right)\text{or}\;\log\left(\frac{P_{n\left(\text{Disagree}\right)}}{P_{n\left(\text{Agree}\right)}}\right)\;\text{or}\;\\ \log\left(\frac{P_{n\left(\text{Agree}\right)}}{P_{n\left(\text{Strongly}\;\text{Agree}\right)}}\right) \end{array}\right]\\ =w_{m}(\theta_{n}+\delta_{1}{\left(\text{Type}0\right)}_{i}+\delta_{2}{\left(\text{Type}1\right)}_{i}+\tau_{11}{\left(\text{Type}0\right)}_{i}+\tau_{21}{\left(\text{Type}1\right)}_{i}\\ +\tau_{12}{\left(\text{Type}0\right)}_{i}+\tau_{22}{\left(\text{Type}1\right)}_{i}+\varepsilon_{j}) \end{array}$$

Equation 5 can be explained in the same manner as Equation 4, only with an addition of a person-level covariate *Country Type*. The parameter $\delta_{3}$ estimates the location of first threshold for *Country Type: 1*, which is the effect of Western/individualist cultural orientations on students’ attitudes towards collaboration compared with the Eastern/collectivist culture.


(5) $$\begin{array}{l} w_{m}\left[\log\left(\frac{P_{n\left(\text{Strongly Disagree}\right)}}{P_{n\left(\text{Disagree}\right)}}\right)or\;\log\left(\frac{P_{n\left(\text{Disagree}\right)}}{P_{n\left(\text{Agree}\right)}}\right)\;\text{or}\;\log\left(\frac{P_{n\left(\text{Agree}\right)}}{P_{n\left(\text{Strongly Agree}\right)}}\right)\right]\\ =w_{m}(\theta_{n}+\delta_{1}{\left(\text{Type}0\right)}_{i}+\delta_{2}{\left(\text{Type}1\right)}_{i}+\delta_{3}{\left(\text{CountryType}1\right)}_{n}\\ +\tau_{11}{\left(\text{Type}0\right)}_{i}+\tau_{21}{\left(\text{Type}1\right)}_{i}+\tau_{12}{\left(\text{Type}0\right)}_{i}+\tau_{22}\left(\text{Type}1{)}_{i}+\varepsilon_{j}\right) \end{array}$$

Equation 6 includes an interaction term *Item Type: valuing teamwork* * *Country Type: Western* to further examine whether there is interaction effect between students’ cultural orientations and their attitudes towards two sub types of collaboration.


(6) $$\begin{array}{l} w_{m}\left[\;\log\left(\frac{P_{n\left(\text{Strongly Disagree}\right)}}{P_{n\left(\text{Disagree}\right)}}\right)\text{or}\;\log\left(\frac{P_{n\left(\text{Disagree}\right)}}{P_{n\left(\text{Agree}\right)}}\right)\;\text{or}\;\log\left(\frac{P_{n\left(\text{Agree}\right)}}{P_{n\left(\text{Strongly Agree}\right)}}\right)\right]\\ =w_{m}(\theta_{n}+\delta_{1}{\left(\text{Type}0\right)}_{i}+\delta_{2}{\left(\text{Type}1\right)}_{i}+\delta_{3}{\left(\text{CountryType}1\right)}_{n}\\ +\delta_{4}{\left(\text{Type}1\times \text{CountryType}1\right)}_{n}+\tau_{11}{\left(\text{Type}0\right)}_{i}+\tau_{21}{\left(\text{Type}1\right)}_{i}\\ +\tau_{12}{\left(\text{Type}0\right)}_{i}+\tau_{22}\left(\text{Type}1{)}_{i}+\varepsilon_{j}\right) \end{array}$$

Equations 7, 8 examine the effect of *Item Type* and *Country ID*, with and without interactions, on students’ attitudes of collaboration. In both equations, China (*Country ID: 1*) was set as the baseline for comparisons.


(7) $$\begin{array}{l} w_{m}\left[\log\left(\frac{P_{n\left(\text{Strongly Disagree}\right)}}{P_{n\left(\text{Disagree}\right)}}\right)\text{or}\;\log\left(\frac{P_{n\left(\text{Disagree}\right)}}{P_{n\left(\text{Agree}\right)}}\right)\;\text{or}\;\log\left(\frac{P_{n\left(\text{Agree}\right)}}{P_{n\left(\text{Strongly Agree}\right)}}\right)\right]\\ =w_{m}(\theta_{n}+\delta_{1}{\left(\text{Type}0\right)}_{i}+\delta_{2}{\left(\text{Type}1\right)}_{i}+\delta_{3}{\left(\text{Country}2\right)}_{n}\\ +\delta_{4}{\left(\text{Country}3\right)}_{n}+\delta_{5}{\left(\text{Country}4\right)}_{n}+\delta_{6}{\left(\text{Country}5\right)}_{n}\\ +\tau_{11}{\left(\text{Type}0\right)}_{i}+\tau_{21}{\left(\text{Type}1\right)}_{i}+\tau_{12}{\left(\text{Type}0\right)}_{i}+\tau_{22}\left(\text{Type}1{)}_{i}+\varepsilon_{j}\right) \end{array}$$

(8) $$\begin{array}{l} w_{m}\left[\log\left(\frac{P_{n\left(\text{Strongly Disagree}\right)}}{P_{n\left(\text{Disagree}\right)}}\right)\text{or}\;\log\left(\frac{P_{n\left(\text{Disagree}\right)}}{P_{n\left(\text{Agree}\right)}}\right)\;\text{or}\;\log\left(\frac{P_{n\left(\text{Agree}\right)}}{P_{n\left(\text{Strongly Agree}\right)}}\right)\right]\\ =w_{m}(\theta_{n}+\delta_{1}{\left(\text{Type}0\right)}_{i}+\delta_{2}{\left(\text{Type}1\right)}_{i}+\delta_{3}{\left(\text{Country}2\right)}_{n}\\ +\delta_{4}{\left(\text{Country}3\right)}_{n}+\delta_{5}{\left(\text{Country}4\right)}_{n}+\delta_{6}{\left(\text{Country}5\right)}_{n}\\ +\delta_{8}{\left(\text{Type}1\times \text{Country}2\right)}_{ni}+\delta_{9}{\left(\text{Type}1\times \text{Country}3\right)}_{ni}\\ +\delta_{10}{\left(\text{Type}1\times \text{Country}4\right)}_{ni}+\delta_{11}{\left(\text{Type}1\times \text{Country}5\right)}_{ni}\\ +\tau_{11}{\left(\text{Type}0\right)}_{i}+\tau_{21}{\left(\text{Type}1\right)}_{i}+\tau_{12}{\left(\text{Type}0\right)}_{i}+\tau_{22}\left({\left(\text{Type}1\right)}_{i}+\varepsilon_{j}\right) \end{array}$$

#### Data analysis procedures

The present study utilized the *eirm* package (Bulut, 2021), which is an extension of the *lme4* package (Bates et al., 2015) in R. Before estimating the models, polytomous item responses were transformed into a pseudo-polytomous form using the *polyreformat* function in the *eirm* package given the link function underlying the explanatory IRT models is the logit function with a binomial distribution. Table 3 demonstrates the transformation of the original polytomous to pseudo-polytomous responses.

**Table 3:** Transformed survey responses for EPCM.

Original response	Category “Disagree”	Category “Agree”	Category “Strongly Agree”
Strongly Disagree	0	NA	NA
Disagree	1	0	NA
Agree	NA	1	0
Strongly Agree	NA	NA	1

The five EIRMs were not completely nested within each other. Therefore, we compared the models based on the relative fit indices of Akaike Information Criterion (AIC; Akaike, 1974) and Bayesian Information Criterion (BIC; Schwarz, 1978). Both AIC and BIC are relative fit indices penalized by the number of parameters. The smaller AIC and BIC values are, the better the model fits the data.

## Results

### Psychometric properties of items in the collaboration scale

In PCM, the locations of the first threshold parameters “*Strongly Disagree/Disagree*” can be interpreted as item easiness. That is, the higher the estimate is, the easier it is to endorse the item for students. Generally, the easiness of all thresholds of the eight items are within [−3, 3], suggesting the items in the two scales are set appropriately with no invalid items. Table 4 presents the estimates of the first threshold parameters (δ) and the distances between the first threshold and the subsequent parameters (τ) for all items in the Collaboration Scale. Results show that students from the selected countries are least likely to choose to *Disagree* over *Strongly Disagree* with the statement “*I prefer working as part of a team to working alone*” (Item1:1.94 logits), followed by “*I find that teamwork raises my own efficiency*” (Item7:2.25 logits) and “*I enjoy cooperating with peers*” (Item8:2.34 logits) among all items based on the *Strongly Disagree/Disagree* threshold. In the contrast, students are most likely to choose to *Disagree* over *Strongly Disagree* with the statement “*I am a good listener*” (Item2:2.90 logits), followed by “*I take into account what others are interested in*” (Item4:2.88 logits) and “*I enjoy considering different perspectives*” (Item6:2.85 logits).

**Table 4:** Locations of “Strongly Disagree/Disagree,” “Disagree/Agree” and to “Agree/Strongly Agree” thresholds for the partial credit model.

	Threshold of “*Strongly Disagree/Disagree*”		Distance to “*Disagree/Agree*”		Distance to “*Agree/Strongly Agree*”	
	Estimate	SE	Estimate	SE	Estimate	SE
Item1	1.94	0.03	1.06	0.02	−1.40	0.02
Item2	2.90	0.06	2.16	0.02	−1.24	0.02
Item3	2.43	0.05	2.28	0.02	−1.29	0.02
Item4	2.88	0.06	2.09	0.02	−1.63	0.02
Item5	2.49	0.04	1.42	0.02	−1.09	0.02
Item6	2.85	0.05	1.94	0.02	−1.23	0.02
Item7	2.25	0.03	1.12	0.02	−1.36	0.02
Item8	2.34	0.05	2.25	0.02	−0.81	0.02

All the results are significant at 0.001 level. SE, standard error.

The distances to *Disagree/Agree* categories for the eight items vary from 1.06 to 2.25 logits, which shows the eight items within collaboration scales have different distances from *Disagree* to *Agree*. Item 1 (1.06 logits) and Item 7 (1.12 logits) have the smallest distances from *Disagree* to *Agree*, indicating it is more difficult for students to change their attitudes from *Disagree* to *Agree* on the statements “*I prefer working as part of a team to working alone*” and “*I find that teamwork raises my own efficiency*.” However, Item 3 and Item 8 have the larger distances between *Disagree* and *Agree* categories, indicating that it is easier to raise students’ levels of agreement on the statement of “*I enjoy seeing my classmates be successful*” and “*I enjoy cooperating with peers*”.

It is noted that the distances between *Agree* and *Strongly Agree* categories are all negative values ranging from −1.63 to −0.81. PCM does not require strict ordering of response categories and thus it allows researchers to estimate threshold parameters that may not follow the same order as the response categories. The negative threshold parameters suggest that the thresholds are not increasingly ordered as the response categories, suggesting it is harder for students to choose *Strongly Agree* category to *Agree* category compared with other thresholds. Additionally, the results from PCM demonstrate that students generally hold positive attitudes towards collaboration, it is easier for them to choose the response category *Strongly Disagree* over *Disagree* compared with the other thresholds.

### Explanatory partial credit model

Table 5 summarizes the results estimated by the five explanatory models using EPCMs. As explained earlier, the explanatory models included different item-level covariates, person-level covariates, and their interactions. Relative fit indices are presented at the bottom of Table 5 to facilitate the comparison of non-nested models.

**Table 5:** Summary of the five EPCM results.

	Model 1		Model 2		Model 3		Model 4		Model 5	
	Est	SE	Est	SE	Est	SE	Est	SE	Est	SE
Random Effects (Variance)	0.68		0.68		0.58		0.58		0.58	
Itemtype0 (Valuing Relationships)	2.39	0.02	2.39	0.02	2.21	0.02	2.63	0.03	2.40	0.03
Itemtype1 (Valuing Teamwork)	2.49	0.02	2.48	0.02	2.36	0.02	2.72	0.03	2.67	0.03
CountryType: Western			0.06	0.01	0.15	0.01				
Itemtype1×CountryType: Western					−0.16	0.02				
CountryID: Korea							−0.20	0.03	−0.12	0.03
CountryID: Japan							−0.43	0.03	−0.30	0.03
CountryID: Canada							−0.24	0.02	−0.05	0.03
CountryID: US							−0.19	0.03	−0.03	0.03
Itemtype1×CountryID: Korea									−0.16	0.04
Itemtype1×Country ID: Japan									−0.09	0.04
Itemtype1×CountryID: Canada									−0.29	0.03
Itemtype1×CountryID: US									−0.25	0.04
“*Strongly Disagree/Disagree*” to “*Disagree/Agree*” to “*Agree/Strongly Agree*” – Step × ItemType										
Itemtype0: PCMcategorycat_3	−0.51	0.02	−0.51	0.02	−0.47	0.02	−0.53	0.02	−0.48	0.02
Itemtype1: PCMcategorycat_3	−0.77	0.02	−0.77	0.02	−0.75	0.02	−0.79	0.02	−0.77	0.02
Itemtype0: PCMcategorycat_4	−3.80	0.02	−3.80	0.02	−3.64	0.02	−3.82	0.02	−3.65	0.02
Itemtype1: PCMcategorycat_4	−3.56	0.02	−3.56	0.02	−3.43	0.02	−3.59	0.02	−3.45	0.02
AIC	831,150		831,149		831,148		831,146		831,142	
BIC	307,544		307,524		307,453		307,288		307,205	
*df*	307,625		307,617		307,557		307,416		307,379	

All the results are significant at 0.001 level. Est, estimate; SE, standard error; df, degree of freedom.

#### Effect of item type

The results of Model 1 show that the items measuring valuing teamwork ($\delta_{\text{Teamwork}}=2.49$) are easier to endorse than the items measuring valuing relationships ($\delta_{\text{Relationship}}=2.39$) by students from all selected countries on choosing *Disagree* over *Strongly Disagree*. However, students are more likely to choose to *Agree* over *Disagree* on the items related to valuing relationships ($\tau_{\text{Relationship}\_1}=-.51$) compared with valuing teamwork ($\tau_{\text{Teamwork}\_1}=-.77$), which is in the reversed order compared with the first threshold location. For the final thresholds, the items related to valuing teamwork ($\tau_{\text{Teamwork}\_2}=-3.56$) are easier to endorse compared with the items related to valuing relationships ($\tau_{\text{Relationship}\_2}=-3.80$). The results show that a higher level of endorsement on collaboration is necessary to strongly agree on valuing relationships than valuing teamwork, regardless of the country effects.

#### Effect of item type and cultural orientations

The second model examines the effects of both item type and country type (Western/Individualist vs. Eastern/Collectivist) on the levels of endorsing collaboration items. The results concerning item type are like those of Model 1. Regarding person-level covariate, the main effect of *Country Type*: *Western* shows that there is a minor yet significant positive effect on Western countries ($\delta_{\text{Western}}=0.06$, *SE* = 0.01, *p* < 0.001) compared with Eastern countries. That is, students from Western countries are $e^{0.06}$= 1.06 times more likely to endorse with collaboration than students from Eastern cultures.

Model 3 introduces an interaction term between item type and country type in addition to the main effects of these covariates. In Model 3, it is easier to agree on the items on valuing teamwork than valuing relationships, and the effect on *Country Type*: *Western* increases to $\delta_{\text{Western}}=0.15$, which suggests that students from Western countries are $e^{0.15}$= 1.16 times more likely to endorse with collaboration than students from Eastern countries.

Besides main effects, there is a negative effect on the interaction term ($\delta_{\text{Teamwork}*\text{Western}}=-0.16$, *SE* = 0.02, *p* < 0.001). The results indicate that compared with Eastern cultures, students from Western cultures are less likely to endorse with valuing teamwork than valuing relationships. In both models that incorporated item-level and person-level covariates, the interactions between item type and the distances from the first threshold parameter show a similar pattern in Model 1 that it requires a higher level of endorsement on collaboration to choose to *Agree* over *Disagree* on the items related to valuing teamwork and *Strongly Agree* over *Agree* on the items related to valuing relationships.

#### Effect of item type and country identities

Models 4 and 5 examine item-level and person-level covariates including item type and country ID. The main effects of item type in the two models are similar to the previous models. Model 4 suggest that when setting China as the baseline of comparison, there are negative effects on all the selected countries, including Korea ($\delta_{\text{Korea}}=-0.20$, *SE* = 0.03, *p* < 0.001), Japan ($\delta_{\text{Japan}}=-0.43$, *SE* = 0.03, *p* < 0.001), Canada ($\delta_{\text{Canada}}=-0.24$, *SE* = 0.02, *p* < 0.001), and the US ($\delta_{US}=-0.19$, *SE* = 0.03, *p* < 0.001).

Furthermore, among the countries, students from Japan hold the most negative attitudes towards collaboration, followed by Canada, Korea, and the United States. Therefore, we concluded that students from China are most likely to value collaboration among all the selected countries.

Model 5 provides more information on how students from different countries differ between valuing relationships and valuing teamwork. Results from Model 5 show that compared with China, there are negative effects on the United States ($\delta_{US}=-0.03$, *SE* = 0.03, *p* < 0.001), Canada ($\delta_{\text{Canada}}=-0.05,$ *SE* = 0.02, *p* < 0.001), Korea ($\delta_{\text{Korea}}=-0.12$, *SE* = 0.03, *p* < 0.001), and Japan ($\delta_{\text{Japan}}=-0.30$, *SE* = 0.03, *p* < 0.001) with increasing order. Also, results suggest that compared with China, Korea ($\delta_{\text{Teamwork}*\text{Korea}}=-0.16$, *SE* = 0.04, *p* < 0.001), Japan ($\delta_{\text{Teamwork}*\text{Japan}}=-0.09$, *SE* = 0.04, *p* < 0.001), Canada ($\delta_{\text{Teamwork}*\text{Canada}}=-0.29$, *SE* = 0.03, *p* < 0.001) as well as the United States ($\delta_{\text{Teamwork}*US}=-0.25$, *SE* = 0.04, *p* < 0.001) are less likely to value teamwork than relationships.

## Discussion and conclusion

The present study used the EIRM approach to examine the impact of cultural orientations on students’ attitudes towards collaboration. There are several trends in the findings. First, students from all countries generally endorsed valuing teamwork more than valuing relationships. Second, students from Western countries with individualist cultures are more likely to value collaboration than Eastern countries with collectivist cultures. Third, China has the highest endorsement of collaboration, followed by the United States, Canada, Korea, and Japan. However, based on the CPS assessment scores, Japan has the highest scores, followed by Korea, Canada, the United States, and China, which is in the exactly reversed order of their endorsement with collaboration. Therefore, we concluded that the two hypotheses of the present study are rejected that (1) students from collectivist cultures are not ensured to have a higher endorsement of collaboration compared with students from individualist cultures and (2) higher endorsement of collaboration does not necessarily lead to higher achievement in the CPS assessment.

Findings from the present study were compared with previous studies on attitudinal differences in collaboration among various cultural orientations. In Hofstede’s longitudinal survey on 76 countries’ inclinations to collaboration, they proposed a novel paradigm to model their positions on the collaboration dimension of the societies (Hofstede, 2011). The results reveal that the pattern differences between Western and Eastern countries are polarized. Generally, individualism tends to prevail in developed and Western countries, with the United States having the highest IDV score. On the other hand, collectivism prevails in less developed and Eastern countries, whereas Japan takes a middle position on this dimension. Hofstede et al. attributed this distribution of countries’ values mainly to their wealth.

PISA 2015 provides a new window to observe middle school students’ attitudes towards collaborative problem-solving in two folds—valuing teamwork and valuing relationships, which reveals two dimensions of collaboration instead of regarding it as a single factor. In addition to attitudes, PISA 2015 also included a collaborative problem-solving assessment to measure students’ performance on CPS tasks based on some real-life scenarios. Findings from the current study revealed that students from Eastern countries did not necessarily have a higher endorsement of collaboration as indicated in the previous cross-national surveys. Instead, only China demonstrated a strong representation of collectivist culture and showed the highest levels of endorsement with collaboration. In contrast, Japan and Korea showed the least agreement on items related to collaboration among the five selected countries.

An unexpected finding of the present study revealed that valuing collaboration did not lead to better success on CPS performance in PISA 2015. Countries with lower endorsement of collaboration demonstrated better performance on the CPS assessment. Previous studies on the relationship between attitude and achievement held different positions. Some studies show that attitude is a positive predictor of achievement (e.g., Lupo et al., 2017; Graham et al., 2007), while others claim attitude does not ensure greater achievement (e.g., Martin and Rainey, 1993). Findings from the present study indicate the gap between students’ attitudes shaped by cultural background and achievement based on schools’ CPS training for students.

### Practical implications

Collaborative problem solving requires cognitive skills and non-cognitive social skills, where attitudes towards collaboration shaped by cultural orientations play a significant role. The present study shed light on understanding the relationship between psychological attributes (i.e., endorsement with collaboration) and performance on collaborative problem solving. Findings revealed that attitudes towards collaboration is not determined by national culture. Further, CPS is not simply determined by psychological characteristics like attitudes. Future research is necessary to understand and address the pedagogical and instructional gaps in CPS training among different countries, after accounting for the effects of confounding factors such as cultural orientations.

### Limitation

Some limitations were identified. First, the present study selected limited countries to represent the Western and Eastern cultures. Future research could be conducted among more countries and regions for global comparisons. Second, the sample selection and data analyses in the present study used country as the minimum unit, whereas overlooking the internal cultural variation within large countries like China, the US, and Canada. Further inspections could be conducted to investigate the differences of attitudes towards collaborations within countries.

## Data Availability

The original contributions presented in the study are included in the article/supplementary material, further inquiries can be directed to the corresponding author.
